# Maintenance of chondrocyte phenotype during expansion on PLLA microtopographies

**DOI:** 10.1177/2041731418789829

**Published:** 2018-08-06

**Authors:** Elisa Costa, Cristina González-García, José Luis Gómez Ribelles, Manuel Salmerón-Sánchez

**Affiliations:** 1 Centre for Biomaterials and Tissue Engineering (CBIT), Universitat Politècnica de València, Valencia, Spain; 2 Biomedical Research Networking Center in Bioengineering, Biomaterials and Nanomedicine (CIBER-BBN), Valencia, Spain; 3 Centre for the Cellular Microenvironment, University of Glasgow, Glasgow, UK

**Keywords:** Poly(L-lactic acid), chondrocyte dedifferentiation, cell adhesion, integrins

## Abstract

Articular chondrocytes are difficult to grow, as they lose their characteristic phenotype following expansion on standard tissue culture plates. Here, we show that culturing them on surfaces of poly(L-lactic acid) of well-defined microtopography allows expansion and maintenance of characteristic chondrogenic markers. We investigated the dynamics of human chondrocyte dedifferentiation on the different poly(L-lactic acid) microtopographies by the expression of collagen type I, collagen type II and aggrecan at different culture times. When seeded on poly(L-lactic acid), chondrocytes maintained their characteristic hyaline phenotype up to 7 days, which allowed to expand the initial cell population approximately six times without cell dedifferentiation. Maintenance of cell phenotype was afterwards correlated to cell adhesion on the different substrates. Chondrocytes adhesion occurs via the *α*_5_*β*_1_ integrin on poly(L-lactic acid), suggesting cell–fibronectin interactions. However, *α*_2_*β*_1_ integrin is mainly expressed on the control substrate after 1 day of culture, and the characteristic chondrocytic markers are lost (collagen type II expression is overcome by the synthesis of collagen type I). Expanding chondrocytes on poly(L-lactic acid) might be an effective solution to prevent dedifferentiation and improving the number of cells needed for autologous chondrocyte transplantation.

## Introduction

Several phenomena account for the inability of articular cartilage to self-repair, such as the avascular nature of the tissue, the limited ability of chondrocytes to migrate into the site of injury, and the absence of mesenchymal cells and a fibrin clot into which cells can migrate.^1,2^ Tissue engineering techniques based on the use of autologous chondrocytes could lead to the regeneration of the damaged cartilage, but it is difficult to obtain sufficient number of cells. This technique involves the biopsy of a small non-bearing site in the joint, the enzymatic digestion of the extracellular matrix (ECM) and isolation of chondrocytes. To increase the number of isolated chondrocytes, cells are cultured in vitro. The major limitation of this technique is chondrocyte dedifferentiation during expansion in monolayer culture: the phenotype of chondrocytes is unstable, and they dedifferentiate into fibroblast-like cells, collagen type II is down regulated during cell division, and in return, cells start producing collagen type I and changing the pattern of proteoglycan synthesis.^3–6^ As a consequence, chondrocytes expanded in vitro do not maintain their characteristic phenotype and, consequently, their ability to regenerate damaged cartilage is impaired. Dedifferentiated chondrocytes do not synthesize the adequate ECM, instead, a fibrous tissue containing collagen type I is produced, which is not able to resist the external mechanical solicitations.

Even though a broad set of substrates have been investigated, chondrocyte dedifferentiation during expansion in monolayer culture has not been solved yet.^7–11^ The maintenance of chondrocyte phenotype in monolayer culture is usually achieved by keeping their characteristic rounded-like morphology. Chondrocytes grown on collagen type II maintained their phenotype for more than two weeks in vitro, but the number of cells decreased as a function of time.^12^ Culturing at high cell densities has also been shown to be an efficient method to avoid chondrocyte dedifferentiation. After 21 days in Petri dish culture, an extensive ECM of collagen type II was produced, but lacking any cell proliferation.^13,14^

The re-differentiation of dedifferentiated human chondrocytes obtained after monolayer expansion has been investigated as a source to get enough cells for autologous chondrocyte transplantation. Several strategies have been proposed, which include the growth of dedifferentiated chondrocytes from monolayer passages P1-P4 in high-density cultures^15^ and then re-seeding dedifferentiated chondrocytes on plasma-treated polymers in both serum-containing and serum-free media.^16^ The dedifferentiation of chondrocytes in culture is usually associated with changes in cell morphology, from a rounded to a spread one. However, cell morphology is not always linked to phenotype.^5^ It has been shown that the loss of hallmarks for differentiated chondrocytes – chondroitin sulphate proteoglycan (CSPG) and collagen type II expression – is coincident with the presence of well-defined F-actin cables within the cytoplasm.^17^ Moreover, chemically induced dedifferentiated chondrocytes re-express collage type II and aggregating proteoglycans, after modifying actin filaments with dihydrocytochalasin B to disassemble actin filaments in the cell periphery.^18,19^

We have shown the absence of F-actin fibres on chondrocytes expanded on poly(L-lactic acid) (PLLA) substrates, suggesting that cells are able to adhere and proliferate on PLLA, for up to 7 days, keeping their characteristic phenotype.^20^ Chondrocytes grown in postconfluence, that is, the ones that are forced to grow on an already existing layer of cells dedifferentiate, as proved by the reorganization of F-actin cytoskeleton.^20^

Here, we investigate the dynamics of chondrocyte dedifferentiation on PLLA substrates by following collagen type I, collagen type II and aggrecan expression as a function of time. A model is proposed in which chondrocytes adhere on PLLA through the *α*_5_*β*_1_ integrin, characteristic for chondrocyte–fibronectin (FN) interaction. However, chondrocytes adhere via the *α*_2_*β*_1_ integrin on the control surface to cell-secreted collagen type I, speeding up their dedifferentiation process.

## Materials and methods

### Materials

PLLA was synthesized by classical polycondensation procedures. Briefly, a glass polymerization reactor, equipped with a nitrogen flow-through inlet and a vacuum connection, was placed in a temperature-controlled bath containing silicone oil. Polymerization was performed in a nitrogen atmosphere at a temperature range of 100°C–150°C for 12–48 h. In order to remove residual monomers, chloroform and methanol were used as solvent and precipitant, respectively. The molecular weights of the polymer, M_n_ and M_w_, were 58,000 and 132,000, respectively, evaluated by gel permeation chromatography (Shimadzu, LC 10A, Japan) using polystyrene (PS) as standard and chloroform as solvent. Samples for atomic force microscopy (AFM) and cell culture were casted from a 1 wt% solution in chloroform on Petri dishes and afterward cut in circular shapes (*φ* = 13 mm). The thickness of the polymer layer was around 5 µm, estimated from the weight of the sample and the PLLA density.

### Substrates

The thermal treatments started with annealing the sample for 2 min at 200°C, it was then cooled to the crystallization temperature, 110°C and maintained for 2 h. A set of samples was prepared by quenching from the melt to room temperature to obtain an amorphous-smooth sample. Surfaces of the samples are highly reproducible (for the same thermal treatment) as checked by AFM.

### Chondrocyte isolation

Human articular cartilage from the knee of a patient undergoing total knee arthroplasty was processed for chondrocyte isolation. Briefly, the cartilage tissue was aseptically dissected from the joint, minced, and washed with Dulbecco’s modified Eagle’s medium (DMEM; Life Technologies). Then, cartilage was incubated for 30 min with a 0.5 mg/ml hyaluronidase (Sigma-Aldrich) solution and for 1 h with a 1 mg/ml pronase (Merck) solution in a shaking water bath at 37°C. After that, cartilage fragments were washed with DMEM and incubated with a 0.5 mg/ml collagenase-IA (Sigma-Aldrich) solution in a shaking water bath at 37°C overnight. The resulting cell suspension was filtered with a 70-µm cell strainer (BD Biosciences) to remove any undigested tissue, and collagenase was rinsed off with DMEM containing 10% foetal bovine serum (FBS; Invitrogen SA). Finally, the cell suspension obtained was transferred in DMEM supplemented with 10% FBS and 50 µg/ml ascorbic acid (Sigma-Aldrich) to a 75-cm^2^ tissue culture flask (Nunc) and maintained at 37°C, in a humidified atmosphere under 5% CO_2_. The culture medium was replaced every 2 days, and cells were allowed to grow until subconfluence. Then, cells were harvested by trypsinisation and counted with a haemacytometer for experiments on PLLA.

### Cell culture

PLLA films pre-sterilized with 25 kGy gamma radiation were placed in a 24-well tissue culture plate and were soaked in culture medium for 72 h before cell seeding. Then, 1 ml (10^4^ cells) of chondrocytes was placed onto the polymer films and was maintained at 37°C, in a humidified atmosphere under 5% CO_2_ for 1, 3 and 7 days. The culture medium used was DMEM, supplemented with 10% FBS and 50 µg/ml ascorbic acid, and it was renewed every 2–3 days. Each experiment was performed in triplicate per topography and Permanox^®^ slides served as the control substrate.

### Immunofluorescence and cytoskeletal observation

After 1, 3 and 7 days of culture chondrocytes were washed in phosphate-buffered saline (PBS) and fixed in formalin solution (Sigma) at 4°C for 1 h. Afterwards, the samples were rinsed with PBS, and a permeabilising buffer (10.3-g sucrose, 0.292-g NaCl, 0.06-g MgCl_2_, 0.476-g Hepes buffer, 0.5-ml Triton X, in 100 ml of water, pH = 7.2) was added at 4°C for 5 min. In order to reduce the background signal, the samples were then incubated in 1% bovine serum albumin (BSA)/PBS at 37°C for 5 min. Afterwards, samples were incubated in primary antibodies at 37°C for 1 h: rabbit anti-human polyclonal antibody, anti–col-I (1:10 in 1% BSA/PBS, Chemicon International); mouse anti-human monoclonal antibody, anti–col-II (1:50 in 1% BSA/PBS, Chemicon International); mouse anti-human monoclonal antibody, anti-Agg (1:50 in 1% BSA/PBS, Invitrogen); mouse anti-human monoclonal antibody, anti-*β*1 integrin (1:50 in 1% BSA/PBS, Beckman Coulter); mouse anti-human monoclonal antibody, anti-*α*_5_ integrin (1:50 in 1% BSA/PBS, Immunotech); mouse anti-human monoclonal antibody, anti-*α*2 integrin (1:50 in 1% BSA/PBS, Beckman Coulter). Samples were then rinsed in 0.5% Tween 20/PBS three times. Then, Cy3-conjugated rabbit anti-mouse or goat anti-rabbit secondary antibody (1:200 in 1% BSA/PBS, Jackson ImmunoResearch) was added at 37°C for 1 h. Finally, samples were washed before being mounted in Vectashield (Vector Laboratories). A Leica DM6000B fluorescent microscope was used. The image system was equipped with a Leica DFC350FX camera.

## Results

### PLLA substrates

**Figure 1.**
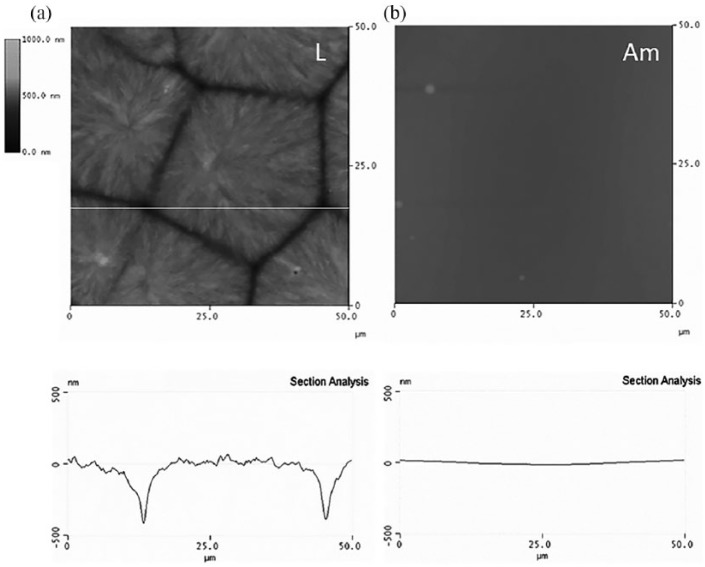
PLLA microtopographies as observed by atomic force microscopy (AFM). Images show the height magnitude using the same scale. A cross-section of each sample is shown at the bottom for the line shown on the figures. (a) Large spherulites (L) and (b) amorphous sample (Am).

Figure 1 shows AFM images (height signal) of PLLA after different thermal treatments, which give rise to surfaces with different microtopographies. A more detail inspection on the effect of thermal history and spherulitic development in PLLA was reported before.^21^ The isothermal crystallization at 110°C after a temperature jump from the melt produces large spherulites, approximately 30–50 µm diameter (Figure 1(a)). The height difference between highest and lowest regions is in the range of 1 µm. By quenching the material from the melt to the glass, amorphous-smooth and flat samples are obtained. These two types of samples will be called hereafter L and Am, respectively, in reference to the size of the spherulites (Large) and to the amorphous character of the sample (Am). The surface roughness for the different samples has been calculated on 50 × 50 µm^2^ (Table 1).

**Table 1.** Roughness parameters of the samples: *R_a_* is the arithmetic average of the height deviations from the centre plane; *R_ms_* is defined as the standard deviation of the height values; and *R_max_* is the difference between the highest and lowest heights. ‘Size’ means the length of the square used to measure roughness.

Sample ID	*R_a_*/nm	*R_ms_*/nm	*R_max_*/µm	Size/µm
L	153.02	188.58	1.01	50
Am	22.58	33.44	0.23	50

L: large spherulites; AM: amorphous sample.

### Chondrocyte dedifferentiation

**Figure 2.**
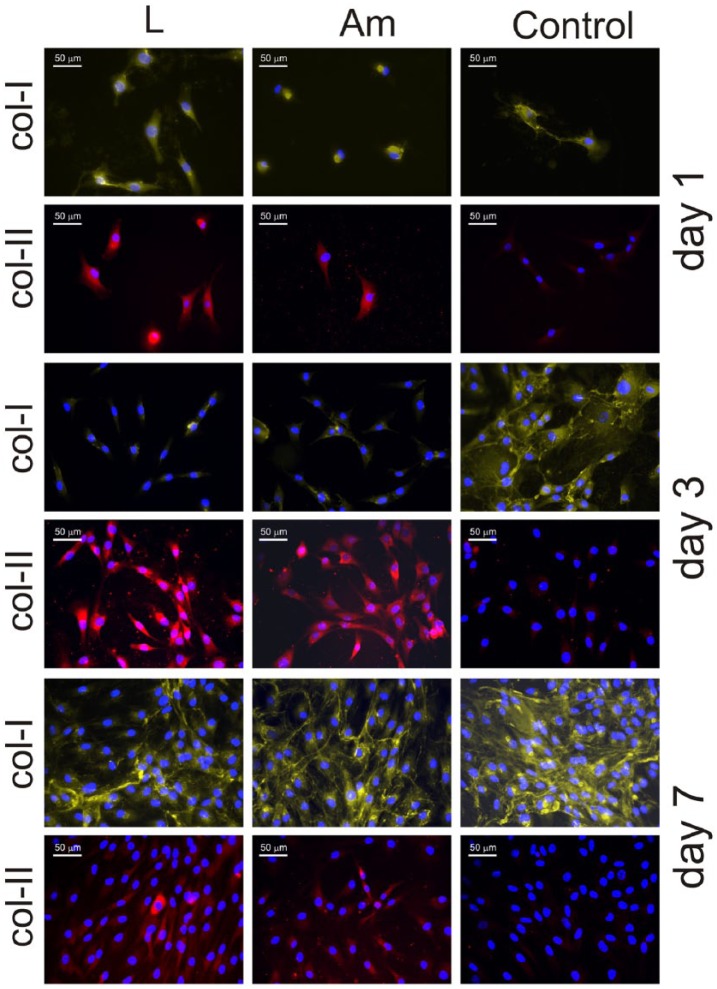
Immunofluorescence for collagen type I (col-I) and collagen type II (col-II) after different culture times (1, 3 and 7 days) on both PLLA (large spherulites and amorphous sample) and the control substrate. Nuclei were counterstained with DAPI.

Figure 2 shows immunofluorescence for collagen type I and collagen type II on the PLLA substrates and the control Permanox after 1, 3 and 7 days. After 1 day of culture, some traces of collagen type I are present on both chondrocytes cultured on PLLA and the control substrate. However, collagen type II is revealed only on those cells adhered on PLLA substrates but not on the control. After 3 days of culture (Figure 2), a well-developed collagen type I ECM has been elaborated by chondrocytes seeded on the control but not on PLLA, which only displays some intracellular collagen type I shadows. As time goes by, collagen type II is maintained on chondrocytes cultured on PLLA, but it disappears completely on the control substrate (Figure 2). After 7 days of culture, the situation is different, the presence of extracellular collagen type I is observed on every substrate, but the density of the network formed is much higher on the control than on PLLA, which shows only some areas of its surface covered by collagen type I fibrils, but still a significant number of collagen type I free cells are observed. Concerning collagen type II, there is no trace of it on the control substrate (only nuclei are observed in Figure 2), but it is clearly depicted on those chondrocytes expanded on PLLA.

**Figure 3.**
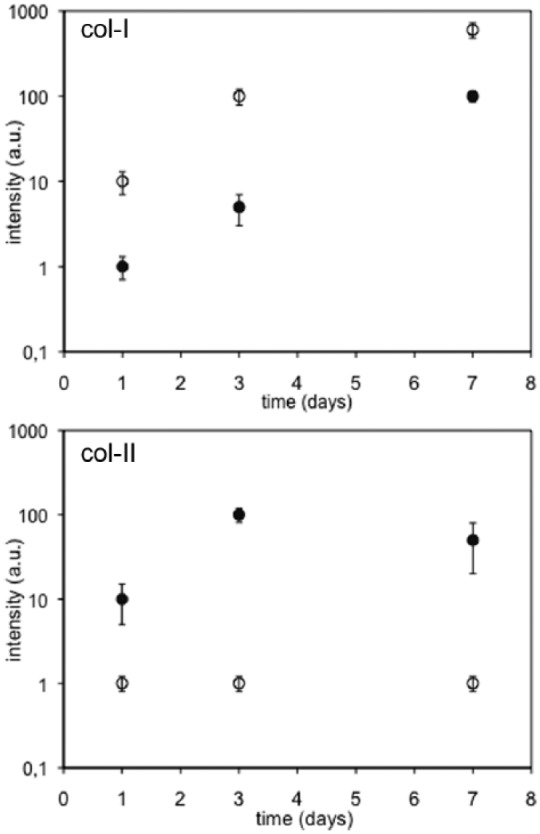
Semi-quantitative analysis for the intensity of the fluorescence to characterize the relative amount of collagen type I (col-I) and collagen type II (col-II) after different culture times on PLLA (open symbols) and the control substrate (filled symbols). The bar is the standard deviation for at least three images.

The temporal sequence shown in Figure 2 can be quantified (in a relative way) by the intensity of the fluorescence as shown in Figure 3. Collagen type I intensity increases at the same rate for cells on both PLLA and the control substrate but with approximately one order of magnitude lower intensity on PLLA. However, collagen type II displays already very low intensity on the control after 1 day of culture, whereas on PLLA, it increases up to the third day of culture and then remains approximately constant up to the seventh day of culture. Simultaneously, cell density increases steadily from 5 × 10^3^ ± 500 (initially) to 30 × 10^3^ ± 800 cells cm^−2^ (day 7), that is, the initial cell population is increased approximately six times.

**Figure 4.**
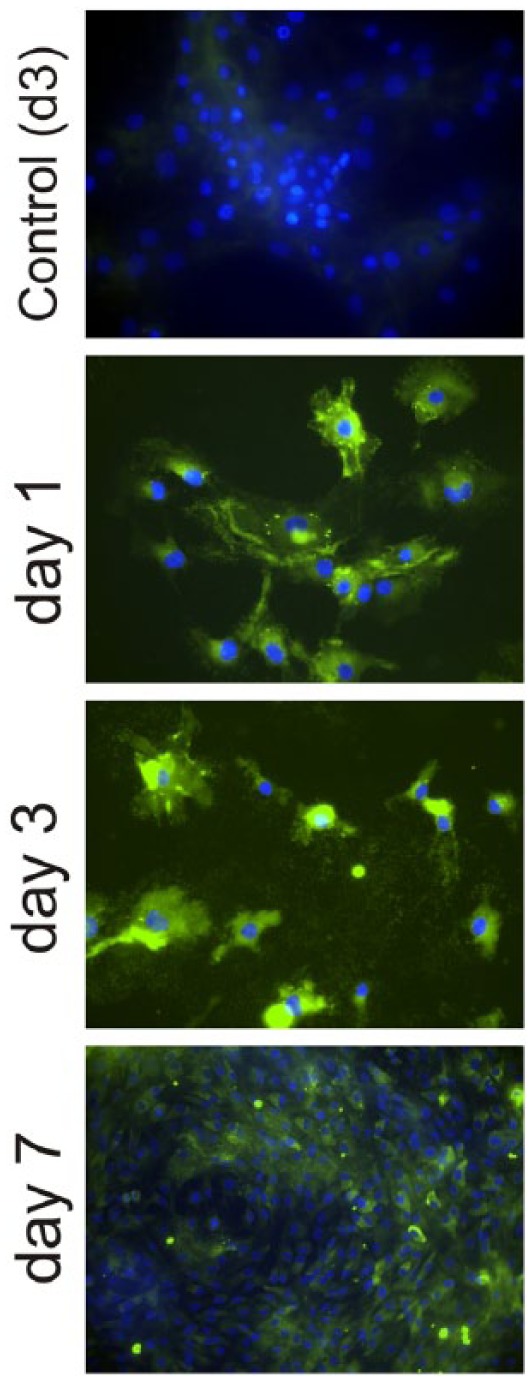
Immunofluorescence for aggrecan after different culture times (1, 3 and 7 days) on both PLLA (L surfaces) and the control substrate (only one picture after 3 days of culture is shown for the control substrate, as no aggrecan was found at any culture time). Nuclei were counterstained with DAPI.

Aggrecan production (Figure 4) was also followed by immunofluorescence both on the control substrate and PLLA. Aggrecan is observed in the ECM after 7 days of culture on PLLA. However, even if there are some aggrecan traces after 1 day of culture, most of it is already completely lost after 3 days of culture on the control permanox (Figure 4 shows cells on the control substrate after 3 days of culture, only nuclei counterstained with 4′,6-diamidino-2-phenylindole (DAPI) can be observed. The picture also represents the situation on the control substrate for 1 and 7 days of culture).

### Cell adhesion

**Figure 5.**
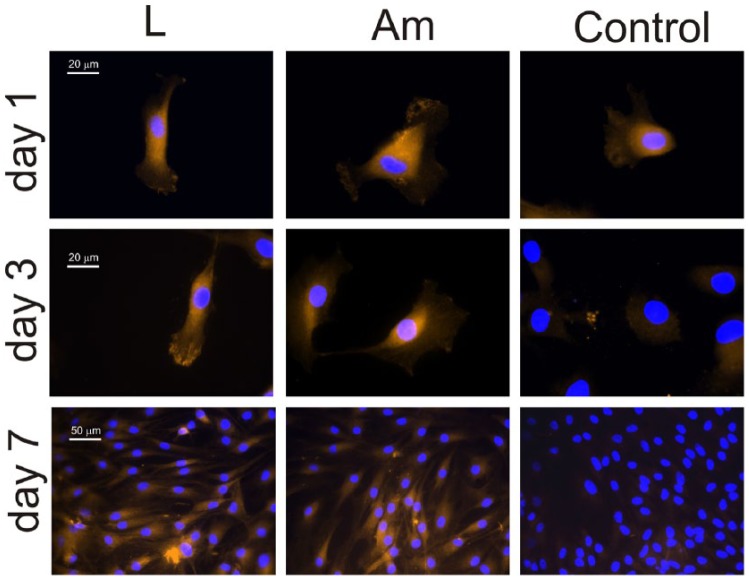
Immunofluorescence for the *α*_5_ integrin after different culture times (1, 3 and 7 days) on both PLLA (large spherulites and amorphous sample) and the control substrate. Nuclei were counterstained with DAPI.

Cell adhesion was studied via immunofluorescence making use of monoclonal antibodies against the *α*_5_, *β*_1_ and *α*_2_ subunits of integrin receptors after different culture times. *α*_5_*β*_1_ integrin mediates interaction with fibronectin, while *α*_2_*β*_1_ is specific for collagen. *α*_5_ integrin is expressed on PLLA after 1, 3 and 7 days (note that for this last culture, time is not evenly distributed for all cells, Figure 5). However, integrin *α*_5_ is observed on the control substrate after 1 day of culture but not after 3 and 7 days (Figure 5).

**Figure 6.**
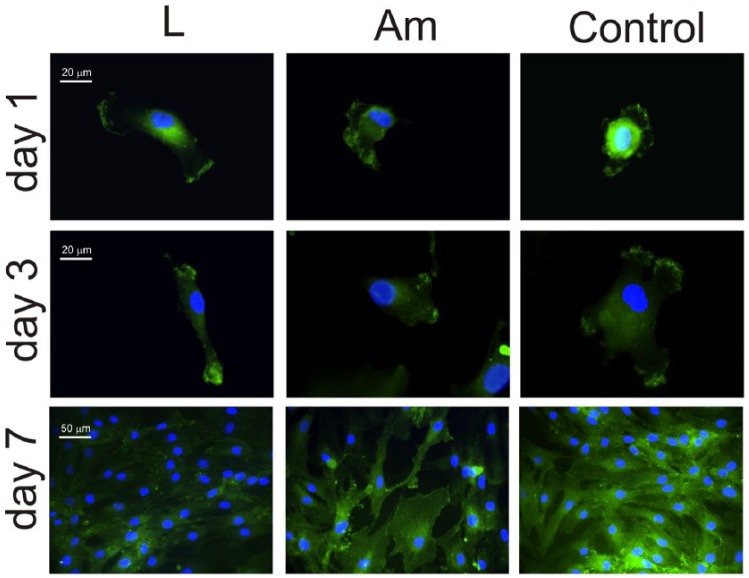
Immunofluorescence for the *β*_1_ integrin after different culture times (1, 3 and 7 days) on both PLLA (large spherulites and amorphous sample) and the control substrate. Nuclei were counterstained with DAPI.

*β*_1_ integrin subunit does follow the trend of *α*_5_ integrin on PLLA substrates, that is, *β*_1_ integrin is expressed on PLLA after 1, 3 and 7 days, evenly distributed throughout the sample. Moreover, unlike *α*_5_, *β*_1_ is also extensively visible on the control substrate for every culture time (Figure 6).

On the other hand, *α*_2_ integrin is expressed on the control substrate after 1, 3 and 7 days of culture; but only after 7 days for cells on PLLA (i.e., there is no *α*_2_ staining after 1 and 3 days of culture on PLLA).

## Discussion

Chondrocyte expansion in monolayer leads to dedifferentiation towards fibroblast-like cells: type I collagen is upregulated, and the pattern of proteoglycan synthesis is modified.^3–6^ Intense research has been done to identify substrates onto which chondrocyte phenotype was maintained; that is, substrates on which chondrocytes can be expanded remaining functional for clinical use afterwards. Chondrocyte culture on different protein monolayers did not alter cell tendency towards dedifferentiation, and signalling cascades were dominated by cell-matrix deposition.^6–11,22–29^ Our results show that even if chondrocytes dedifferentiate on PLLA, the dynamics of the process is slow enough to increase the cell population while collagen type II and aggrecan continue to be the major ECM components (Figures 2 and 3). Furthermore, our results are supported by the lack of F-actin cytoskeleton development after 7 days of chondrocyte culture on PLLA;^20^ whose presence within the cytoskeleton is associated to chondrocyte dedifferentiation.^17^

Chondrocyte morphology is strongly affected by the microtopography of the PLLA substrates.^20^ The presence of microgrooves between well-defined spherulites results in more elongated chondrocytes. Spread cells on amorphous PLLA transform into elongated ones, growing in a characteristic direction and even promoting isolated cells on bigger spherulites (L sample).^20^ However, the dynamics of chondrocyte dedifferentiation is not affected by the underlying microtopography of the substrate (Figure 2): the time evolution for collagen type I and collagen type II follows the same pattern on both big spherulites (L sample) and the amorphous PLLA (Am). This finding supports the idea that changes in cell morphology are not always linked to phenotype expression.^17^

Cell adhesion to the ECM plays a central role in the formation, maintenance and repair of numerous tissues, and it is primarily mediated by the integrin family of adhesion receptors. On synthetic materials, it is usually mediated by ECM proteins, mainly FN, adsorbed onto its surface.^30–32^ The distribution, conformation and strength of adhesion between the protein and the substrate modulate the cell-material interaction.^33–36^ It is well known that fibronectin conformation on a synthetic material depends on surface properties, including chemistry, wettability, hydrophilicity and micro/nano roughness of the sample.^36–39^FN in solution is in a compact-globular conformation.^40,41^ Surfaces of different nature have shown to alter the native globular conformation of the FN molecule upon adsorption, altering its biological activity, especially on slightly charged substrates.^36,39,42^ Our results suggest that FN is adsorbed on PLLA in a conformation that allows chondrocytes to proliferate while maintaining its characteristic, non-differentiated, phenotype until cell confluence is reached. We hypothesize that integrin-mediated adhesion on PLLA, also determines the dynamics of chondrocyte dedifferentiation on monolayer cultures.

The importance of *α*_5_*β*_1_ integrin in maintenance of chondrocyte phenotype has been discussed in the literature. In vivo, lack of expression of the *α*_5_*β*_1_ integrin resulted in hypertrophic chondrocytes.^43^ Chondrocyte adhesion to cartilage, in vitro, is mediated by the *α*_5_*β*_1_ integrin.^44^ Besides, *α*_5_*β*_1_ mediates adhesion, spreading, proliferation and colony formation of chondrocytes; furthermore, it has been proposed that the interaction of *α*_5_*β*_1_ with fibronectin could exert a positive regulatory role in proliferation of chondrocytes in vitro.^45^ *α*_5_*β*_1_ integrin may function as a fundamental chondrocytic mechanoreceptor, most likely through interactions with FN, which may transmit the mechanical forces from the ECM to the cell surface.^46^ On the other hand, chondrocytes synthesize collagen type I on the control substrate. *α*_1_*β*_1_ and *α*_2_*β*_1_ integrins are the major collagen-binding integrins, with *α*_1_*β*_1_ having a higher affinity for the basement membrane collagen type IV and *α*_2_*β*_1_ having a higher affinity for the fibrillar collagen type I.^47–49^

**Figure 7.**
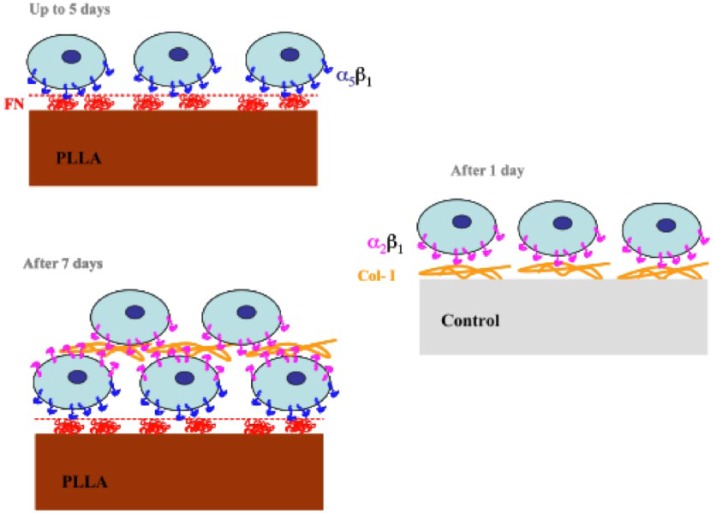
Model for chondrocyte adhesion on PLLA substrates. FN adsorbed in a conformation that enhances *α*_5_*β*_1_ integrin expression which leads to chondrocyte proliferation without dedifferentiation. After confluence, cells are forced to grow on several layers which results in collagen type I production and the loss of the characteristic markers as it happens, with faster dynamics, on the control substrate.

**Figure 8.**
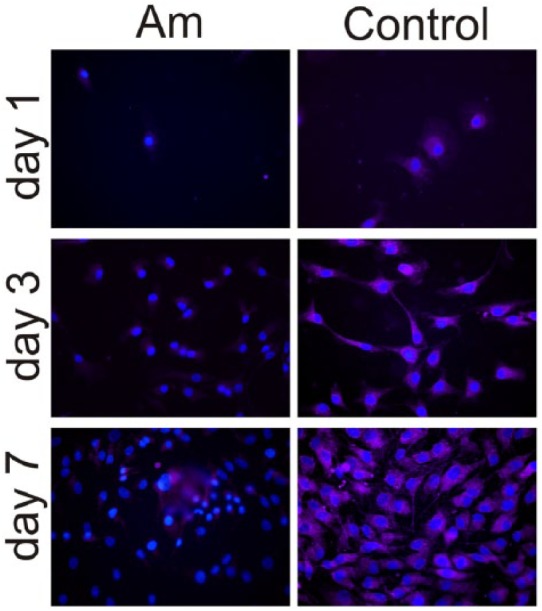
Immunofluorescence for the *α*_2_ integrin after different culture times (1, 3 and 7 days) on both PLLA and the control substrate. Nuclei were counterstained with DAPI.

The biological activity of a substrate depends strongly on the ECM protein that covers its surface before cell interaction. Figure 7 sketches a model for chondrocyte adhesion on PLLA as compared with the control substrate (that accounts for surfaces on which chondrocyte dedifferentiation takes place quickly after cell seeding). Chondrocytes seeded on PLLA interact with FN previously adsorbed on the material substrate (from FBS in the medium) through *α*_5_*β*_1_ integrins (Figures 5 and 6), which leads to cell expansion while maintaining good levels of collagen type II and slow increase in collagen type I production (Figures 2 and 3). After 7 days of culture on PLLA, cell confluence is reached and subsequent proliferation leads to the formation of several cell layers on the material surface. At this point, expression of collagen type I is enhanced (Figure 2) and chondrocytes express receptors for this ECM component, that is, the *α*_2_*β*_1_ integrin turns out to be the main adhesion receptor (Figure 8), which further promotes loss of the characteristic chondrocytic phenotype. On the other hand, when chondrocytes are cultured on the control substrate, FN must be adsorbed in such a conformation that does not enhance *α*_5_*β*_1_ expression at the initial process of the cell–material interaction. Instead, cells start producing collagen type I after a few hours of culture (Figure 2), that immediately turns out to be the main component of the ECM (Figure 2) deposited on the control substrate. Accordingly, cells develop the receptors for collagen type I, the *α*_2_*β*_1_ integrin that further increases the production of the new matrix and the loss of characteristic markers.

## Conclusion

Chondrocyte expansion on PLLA allows the increase in initial cell population approximately six times with collagen type II and aggrecan as the main components of the ECM, which suggests that chondrocytes maintain their characteristic phenotype and proliferate at the same time. This remarkable finding is relevant in clinical applications to repair cartilage starting with the biopsy of a small non-bearing site in the joint, the enzymatic digestion of the ECM, isolation and expansion of chondrocytes before injection in the injured area of the tissue. Moreover, we show that the composition of the ECM during cell expansion, that is, chondrocyte phenotype, is related to the initial cell–material interaction. Chondrocytes seeded on PLLA express *α*_5_*β*_1_ – the main integrin for fibronectin – while secreting aggrecan and collagen type II. Nevertheless, on the control substrate, expression of *α*_2_*β*_1_ – the main receptor for collagen type I – is upregulated which further enhances the secretion of collagen type I and the loss of the chondrocytic phenotype during monolayer expansion.
